# Supplementary material for: Content-rich biological network constructed by mining PubMed abstracts
Source: BMC Bioinformatics. 2004 Oct 8;5:147. doi: 10.1186/1471-2105-5-147 (PMC528731; doi:10.1186/1471-2105-5-147)
Supplement: Additional File 2 — The original results of the above study (non-essential files are deleted to keep the file size under the limit set by BMC bioinformatics). [file 1471-2105-5-147-S2.bz2 › chilibotAdditionalFile2/dip20/658ID10525412E11049/html/NIFD_NIFK.html]

 


 **NIFD** and **NIFK** 
  
Found 195 abstracts in PubMed, retrieved 20.  
 

 What does Google say? 
 PDF only 
| .edu only 

---

**Interactive relationship** (e.g. stimulation, inhibition, etc)

**Non-interactive relationship** (e.g. studied together, co-existance, homology, etc.)

- VnfY is required for full activity of the vanadium containing dinitrogenase  [ **NIFK** ]   [ **NIFD** ]  in Azotobacter vinelandii.  Ref: 12644512 J Bacteriol, 2003
- These results indicate that VnfY has a role in the maturation of the V dependent dinitrogenase  [ **NIFK** ]   [ **NIFD** ] , with a specific role in the formation of the V containing cofactor and or its insertion into apodinitrogenase.  Ref: 12644512 J Bacteriol, 2003
- Certain Azotobacter vinelandii mutant strains unable to synthesize FeMo co accumulate an apo form of dinitrogenase  [ **NIFK** ]   [ **NIFD** ]  lacking FeMo co, with a subunit composition alpha 2 beta 2 gamma 2, which can be activated in vitro by the addition of FeMo co.  Ref: 11823455 J Biol Chem, 2002
- The MoFe protein component of the complex metalloenzyme nitrogenase is an alpha2beta2 tetramer encoded by the  **nifD**  and the  **nifK**  genes.  Ref: 12468552 J Biol Chem, 2003
- In contrast, either GlnB or GlnJ can serve as a critical element in regulation of the reversible ADP ribosylation of dinitrogenase  [ **NIFK** ]   [ **NIFD** ]  reductase catalyzed by the dinitrogenase  [ **NIFK** ]   [ **NIFD** ]  reductase ADP ribosyl transferase DRAT dinitrogenase  [ **NIFK** ]   [ **NIFD** ]  reductase activating glycohydrolase DRAG regulatory system.  Ref: 11591658 J Bacteriol, 2001
- Under salt stress, the amount of dinitrogenase  [ **NIFK** ]   [ **NIFD** ]  reductase inactivated by ADP ribosylation was strongly reduced, indicating that the dinitrogenase  [ **NIFK** ]   [ **NIFD** ]  reductase ADP ribosyl transferase DRAT activity was also inhibited by increased NaCl concentrations.  Ref: 11927988 Curr Microbiol, 2002
- As such this represents a new fold capable of binding FeMo co, where the only previous example was that seen in dinitrogenase  [ **NIFK** ]   [ **NIFD** ] .  Ref: 12754195 J Biol Chem< MedlineTA>J Biol Chem, 2002
- The seven representatives of BOX PCR types produced indole acetic acid, reduced acetylene and showed specific immunological cross reaction with anti dinitrogenase  [ **NIFK** ]   [ **NIFD** ]  reductase antibody.  Ref: 11566385 J Biotechnol, 2001
- in addition, interrelationships between the control of known redox balancing systems, the Calvin Benson Bassham, dinitrogenase  [ **NIFK** ]   [ **NIFD** ]  and dimethyl sulfoxide reductase systems, were probed in strains grown under both photoheterotrophic and photoautotrophic growth conditions.  Ref: 11591679 J Bacteriol, 2001
- The dinitrogenase  [ **NIFK** ]   [ **NIFD** ]  component proteins of the conventional Mo nitrogenase MoFe protein and of the alternative Fe only nitrogenase FeFe protein were both isolated and purified from Rhodobacter capsulatus, redox titrated according to the same procedures and subjected to an EPR spectroscopic comparison.  Ref: 11895435 Eur J Biochem, 2002
- A mutant strain having an insertion mutation in vnfY has 10 fold less vnf dinitrogenase  [ **NIFK** ]   [ **NIFD** ]  activity and exhibits a greatly diminished level of 49 V label incorporation into the V dependent dinitrogenase  [ **NIFK** ]   [ **NIFD** ]  when compared to the wild type.  Ref: 12644512 J Bacteriol, 2003
- Although restriction analysis of the  **nifD**   **nifK**  intergenic DNA fragment confirmed the unique nature of Rhizobium sp.  Ref: 12802480 Arch Microbiol< MedlineTA>Arch Microbiol, 2003
- Dinitrogenase  [ **NIFK** ]   [ **NIFD** ]  is a heterotetrameric alpha 2 beta 2 enzyme that catalyzes the reduction of dinitrogen to ammonium and contains the iron molybdenum cofactor FeMo co at its active site.  Ref: 11823455 J Biol Chem, 2002
- Site directed mutagenesis of the draG gene was used to generate altered forms of dinitrogenase  [ **NIFK** ]   [ **NIFD** ]  reductase activating glycohydrolase DRAG with D123A, H142L, H158N, D243G, and E279R substitutions.  Ref: 11544238 J Bacteriol, 2001
- Supporting this conclusion, we show that strains containing mutations in both nafY and nifX are severely affected in diazotrophic growth and extractable dinitrogenase  [ **NIFK** ]   [ **NIFD** ]  activity when cultured under conditions that are likely to occur in natural environments.  Ref: 11823455 J Biol Chem, 2002
- This regulation is mediated by two enzymes, dinitrogenase  [ **NIFK** ]   [ **NIFD** ]  reductase ADP ribosyl transferase DRAT and dinitrogenase  [ **NIFK** ]   [ **NIFD** ]  reductase activating glycohydrolase DRAG.  Ref: 11315111 Can J Microbiol, 2001
- Mutational analyses suggested that FdxN is an important but not the essential electron donor for dinitrogenase  [ **NIFK** ]   [ **NIFD** ]  reductase.  Ref: 11371540 J Bacteriol, 2001
- Our dinitrogenase  [ **NIFK** ]   [ **NIFD** ]  gene nifH sequences grouped together with a group of cyanobacteria from the subtropical North Pacific.  Ref: 12406777 Appl Environ Microbiol, 2002
- Effects of specific amino acid substitutions on activities of dinitrogenase  [ **NIFK** ]   [ **NIFD** ]  reductase activating glycohydrolase from Rhodospirillum rubrum.  Ref: 11544238 J Bacteriol, 2001
- Samples of the dithionite reduced FeFe protein the dinitrogenase  [ **NIFK** ]   [ **NIFD** ]  component of the Fe only nitrogenase from Rhodobacter capsulatus have been investigated by 57Fe Mössbauer spectroscopy and by Fe and Zn EXAFS as well as XANES spectroscopy.  Ref: 11862539 J Biol Inorg Chem, 2002
- In specific mutant strains of R. capsulatus, expression of both the Calvin Benson Bassham and dinitrogenase  [ **NIFK** ]   [ **NIFD** ]  systems was influenced by dimethyl sulfoxide respiration.  Ref: 11591679 J Bacteriol, 2001
- The DNA isolated from the soil for this purpose was suitable for DNA DNA hybridization using 0.4 0.7 kb probes targeting denitrification enzymes, dinitrogenase  [ **NIFK** ]   [ **NIFD** ]  reductase nifH and eubacterial 16S rRNA.  Ref: 11377771 FEMS Microbiol Ecol< MedlineTA>FEMS Microbiol Ecol, 2001
- As another level of control, nitrogenase activity was regulated by at least three types of mechanisms in strain BH72 covalent modification of dinitrogenase  [ **NIFK** ]   [ **NIFD** ]  reductase NifH, probably by ADP ribosylation, and two other, unknown means.  Ref: 11914357 J Bacteriol, 2002
- These symbionts are thought to provide enzymes, e.g., cellulase and dinitrogenase  [ **NIFK** ]   [ **NIFD** ] , which assist the host in utilizing wood as a primary food source.  Ref: 12450854 Appl Environ Microbiol, 2002
- The Fe only nitrogenase and the Mo nitrogenase from Rhodobacter capsulatus a comparative study on the redox properties of the metal clusters present in the dinitrogenase  [ **NIFK** ]   [ **NIFD** ]  components.  Ref: 11895435 Eur J Biochem, 2002
- Another novel S = 1 2 signal at g = 1.9.92 and 1.77 was observed on further, enzymatic reduction of the dithionite reduced state of Rc1Fe with the dinitrogenase  [ **NIFK** ]   [ **NIFD** ]  reductase component Rc2Fe of the same enzyme system turnover conditions in the presence of N2 and ATP.  Ref: 11895435 Eur J Biochem, 2002
- The key nitrogen fixation nif genes, including nifH,  **nifD** , and  **nifK**  for nitrogenase component proteins as well as nifE, nifN, nifB and nifV for synthesis of the iron molybdenum cofactor FeMoco of nitrogenase, have now been identified in C. acetobutylicum or C. beijerinckii or both.  Ref: 11781802 J Ind Microbiol Biotechnol, 2001
- Significant amounts of both dinitrogenase  [ **NIFK** ]   [ **NIFD** ]  and dinitrogenase  [ **NIFK** ]   [ **NIFD** ]  reductase were only formed when the growth medium was supplemented with molybdate 1 microM.  Ref: 12667075 Biochemistry, 2003
